# Beyond fitness tracking: The use of consumer-grade wearable data from normal volunteers in cardiovascular and lipidomics research

**DOI:** 10.1371/journal.pbio.2004285

**Published:** 2018-02-27

**Authors:** Weng Khong Lim, Sonia Davila, Jing Xian Teo, Chengxi Yang, Chee Jian Pua, Christopher Blöcker, Jing Quan Lim, Jianhong Ching, Jonathan Jiunn Liang Yap, Swee Yaw Tan, Anders Sahlén, Calvin Woon-Loong Chin, Bin Tean Teh, Steven G. Rozen, Stuart Alexander Cook, Khung Keong Yeo, Patrick Tan

**Affiliations:** 1 SingHealth Duke-NUS Institute of Precision Medicine, Singapore; 2 Cancer and Stem Biology Program, Duke-NUS Medical School, Singapore; 3 Cardiovascular and Metabolic Disorders Program, Duke-NUS Medical School, Singapore; 4 National Heart Research Institute Singapore, National Heart Centre Singapore, Singapore; 5 Lymphoma Genomic Translational Laboratory, Division of Medical Oncology, National Cancer Centre Singapore, Singapore; 6 Department of Cardiology, National Heart Centre Singapore, Singapore; 7 Laboratory of Cancer Epigenome, Division of Medical Sciences, National Cancer Centre Singapore, Singapore; 8 Institute of Molecular and Cell Biology, Agency for Science, Technology and Research, Singapore; 9 Cancer Science Institute of Singapore, National University of Singapore, Singapore; 10 Centre for Computational Biology, Duke-NUS Medical School, Singapore; 11 National Heart and Lung Institute, Imperial College London, United Kingdom; 12 MRC Clinical Sciences Centre, Imperial College London, United Kingdom; 13 Biomedical Research Council, Agency for Science, Technology and Research, Singapore; Newcastle University, United Kingdom of Great Britain and Northern Ireland

## Abstract

The use of consumer-grade wearables for purposes beyond fitness tracking has not been comprehensively explored. We generated and analyzed multidimensional data from 233 normal volunteers, integrating wearable data, lifestyle questionnaires, cardiac imaging, sphingolipid profiling, and multiple clinical-grade cardiovascular and metabolic disease markers. We show that subjects can be stratified into distinct clusters based on daily activity patterns and that these clusters are marked by distinct demographic and behavioral patterns. While resting heart rates (RHRs) performed better than step counts in being associated with cardiovascular and metabolic disease markers, step counts identified relationships between physical activity and cardiac remodeling, suggesting that wearable data may play a role in reducing overdiagnosis of cardiac hypertrophy or dilatation in active individuals. Wearable-derived activity levels can be used to identify known and novel activity-modulated sphingolipids that are in turn associated with insulin sensitivity. Our findings demonstrate the potential for wearables in biomedical research and personalized health.

## Author summary

Little is known about how data from wearable sensors can be used apart from fitness tracking. We comprehensively studied 233 normal volunteers, integrating data from wearable sensors with lifestyle questionnaires, cardiac imaging, sphingolipid profiling, and clinical measurements of various heart and metabolic disease markers. Apart from showing that wearable sensors can be used to identify groups of volunteers with distinct behavioral and demographic characteristics, we showed that resting heart rate (RHR) from wearables performed better than step counts in predicting heart and metabolic disease risk markers. Notably, we further demonstrated that wearable data could be used in 2 areas of biomedical research. In the field of cardiac imaging, we showed that activity data from wearables can be used to determine how the size of heart is influenced by physical activity. Wearable data could also identify active individuals that are more likely than others to have enlarged hearts and potentially be misdiagnosed with heart disease. In the field of lipidomics, we showed that wearable data can be used to identify species of sphingolipids that are affected by how active a person is. Some of these compounds are known to be associated with obesity, diabetes, and heart disease.

## Introduction

Public adoption of consumer-grade wearable activity trackers (“wearables”) has been steadily increasing in recent years [1,2], and it is estimated that the global market for wearables will exceed $34 billion US$ by 2020 [3]. Basic activity trackers provide accelerometer-based activity data, whereas more sophisticated models are also capable of monitoring heart rate (HR). Together, these indicators have the potential to provide deep insights into an individual’s cardiovascular health and fitness. For instance, resting heart rate (RHR) is an important indicator of cardiovascular health [4–6], whereas step counts can be used to infer patterns and levels of physical activity. Both metrics play roles in the modulation and prediction of risk of cardiovascular and metabolic disorders (CVMDs) [7].

Given the role played by physical activity in determining health outcomes, there has been great interest in the use of wearables in healthcare. Most research on wearables thus far has been focused on their utility in promoting increased physical activity in healthy and diseased populations [8]. Whereas most studies reported increased physical activity after wearable introduction [8], there is little evidence that this intervention results in clinically significant health outcomes. For instance, a year-long study conducted on corporate employees showed that although wearable introduction increased moderate to vigorous physical activity, it did not improve health outcomes [9]. Furthermore, only around 10% of participants were still using their wearable at study conclusion [9]. More recent studies have also started exploring how wearable data correlate with clinical and biological markers. In one study monitoring wearable data from 43 individuals over an average of 5 months, the authors showed that disease states and physiological differences between individuals (e.g., insulin sensitivity and inflammation) could be discerned from the data [10]. Another study seeking to determine how comprehensive personal data collected from 108 individuals correlated with physiology and disease did not identify any significant correlations with wearable data [11]. There are also studies exploring the use of time series HR data from wearables in the detection of conditions associated with cardiovascular disease such as atrial fibrillation (AF), sleep apnea (SA), and hypertension [12–14]. For example, deep neural networks (DNNs) trained on HR and step count data obtained from the Apple Watch (Apple, www.apple.com) were able to detect AF, SA, and hypertension at accuracies of 97%, 90%, and 82%, respectively [13,14].

Despite these advances, the lack of comprehensive datasets that integrate wearable metrics with other data types means that the utility of consumer-grade wearables to basic, translational, and clinical research, as well as personalized health, remains largely uncharacterized. In this study, our goal was to investigate the utility of consumer-grade wearables in cardiovascular and lipidomics research. To that end, we generated multidimensional data from 233 normal individuals recruited for a longitudinal study (SingHEART/BioBank; National Heart Centre Singapore [NHCS], https://www.nhcs.com.sg). Subjects were tracked using a consumer-grade wearable activity and HR tracker (Fitbit Charge HR; Fitbit, www.fitbit.com), in addition to comprehensive profiling through lifestyle questionnaires, clinical measurements (e.g., weight, height, waist circumference [WC], blood pressure, etc.), lipid panel values, blood glucose test, cardiac magnetic resonance imaging (CMR), and lipidomic profiling. We then performed integrative analysis of the dataset in order to answer three specific questions. First, can wearable metrics obtained from study subjects provide insights into their behavioral and demographic characteristics? Second, how well do wearable metrics (both step- and HR-based) correlate with CVMD risk markers such that they are useful in the areas of clinical and/or translational research and personalized health monitoring? Finally, can wearable-derived metrics be used to support basic research, particularly in the analysis of cardiac imaging and lipidomic profiling data?

## Results

### Characteristics of the volunteer cohort

The cohort of 233 volunteers was tracked for a median duration of 4 days (range 2–6 days) per subject. Summary statistics of this cohort are shown in Table 1 (full details in S1 Data). The cohort had a median age of 48 years (range 21–69 years) and displayed a female bias (137/233, 58.8%). The average daily steps (“DailySteps”) median was 10,395 steps per day, which is consistent with a recent study that compared Fitbit Flex (Fitbit, www.fitbit.com) and ActiGraph (http://actigraphcorp.com/) measurements in 104 Singapore-resident individuals (median steps/day = 10,193) [15]. There was no significant difference in DailySteps between male and female subjects (Student *t* test, *p* = 0.604). In terms of wearable-derived HR metrics, median average day and night HR were 75 bpm and 61 bpm, respectively, with a median RHR of 69 bpm. We compared wearable-derived RHR (denoted henceforth as “RestingHR”) with in-clinic measurements obtained from two sources, namely RHR measured by an automatic blood pressure monitor (ABPM_HR) and during an electrocardiogram test (ECG_HR). We found that RestingHR correlated better with ECG_HR, which is a generally accepted benchmark (r_s_ = 0.690; *p =* 2.506 × 10^−33^), as compared with ABPM_HR (r = 0.541; *p =* 2.434 × 10^−18^). Bland-Altman analysis [16] showed that RestingHR and ABPM_HR were on average higher than ECG_HR (mean difference = 5 bpm and 9 bpm, respectively), with RestingHR having better agreement with ECG_HR (95% limits of agreement = −9 bpm, 20 bpm) compared with ABPM_HR (95% limits of agreement = −12 bpm, 30 bpm). This suggests that RestingHR is relatively accurate and comparable to clinical measurements. Active [10,17] volunteers (DailySteps > 8,000) had lower RestingHR compared with their sedentary counterparts, even after accounting for age, gender, and body mass index (BMI; β = −3.185; *p* = 0.001). This observation was also significant when using either continuous step counts (0.252 bpm less per 1,000 additional steps; *p* = 0.021) or when using self-reported total activity (1.723 bpm less per additional unit; *p* = 4.080 × 10^−6^).

**Table 1:** Summary statistics of volunteers, grouped by gender.

Characteristic	Female (*n* = 137; 58.8%)	Male (*n* = 96; 41.2%)	Test
Age, years	47.49 (11.44)	44.36 (12.63)	0.051
Ethnicity			0.257
Chinese	127 (92.7)	85 (88.5)	
Malay	4 (2.9)	3 (3.1)	
Indian	2 (1.5)	6 (6.2)	
Others	4 (2.9)	2 (2.1)	
BMI, kg/m^2^	22.68 (3.89)	24.65 (3.98)	<0.001
WC, cm	78.33 (10.14)	88.54 (10.88)	<0.001
SBP, mmHg	122.80 (17.36)	133.81 (15.64)	<0.001
DBP, mmHg	72.88 (12.50)	83.12 (11.51)	<0.001
RestingHR, (Fitbit, bpm)	70.37 (6.85)	68.72 (6.80)	0.07
ECG_HR, bpm	64.87 (9.58)	63.45 (11.13)	0.304
Total Cholesterol, mmol/l	5.33 (1.02)	5.26 (0.85)	0.581
LDL, mmol/l	3.28 (0.84)	3.37 (0.92)	0.471
HDL, mmol/l	1.60 (0.34)	1.33 (0.32)	<0.001
TGs, mmol/l	0.98 (0.49)	1.34 (0.88)	<0.001
Glucose, mmol/L	5.24 (0.41)	5.44 (0.64)	0.005
DailySteps, (Fitbit, x1000)	10.74 (4.13)	11.00 (3.66)	0.612
Fitbit ActivityClass			0.799
Cat I	14 (10.2)	10 (10.4)	
Cat II	57 (41.6)	36 (37.5)	
Cat III	54 (39.4)	38 (39.6)	
Cat IV	12 (8.8)	12 (12.5)	
GPPAQ Score	1.25 (1.12)	1.84 (1.15)	<0.001
LVM, g	64.13 (14.49)	93.16 (21.29)	<0.001
LVEDV, ml	107.79 (16.90)	137.36 (25.37)	<0.001
RVEDV, ml	106.21 (19.00)	141.74 (22.65)	<0.001
AoF, ml	65.62 (9.37)	78.39 (12.72)	<0.001

Test *p*-values for between-gender comparisons are shown: For continuous variables, Student *t* test was used, whereas categorical values were evaluated using the chi-squared test. The full dataset is available in S1 Data, and this table was generated by code in S2 Data.

Abbreviations: AoF, aortic forward flow; BMI, body mass index; Cat, category; DailySteps, average daily steps; DBP, diastolic blood pressure; ECG_HR, electrocardiogram heart rate; GPPAQ, General Practice Physical Activity Questionnaire; HDL, high-density lipoprotein; LDL, low-density lipoprotein; LVEDV, left ventricular end-diastolic volume; LVM, left ventricular mass; RestingHR, wearable-derived RHR; RHR, resting heart rate; RVEDV, right ventricular end-diastolic volume; SBP, systolic blood pressure; TG, triglyceride; WC, waist circumference.

### Wearable metrics provide insights on behavioral and demographic stratification of volunteers

**Fig 1:**
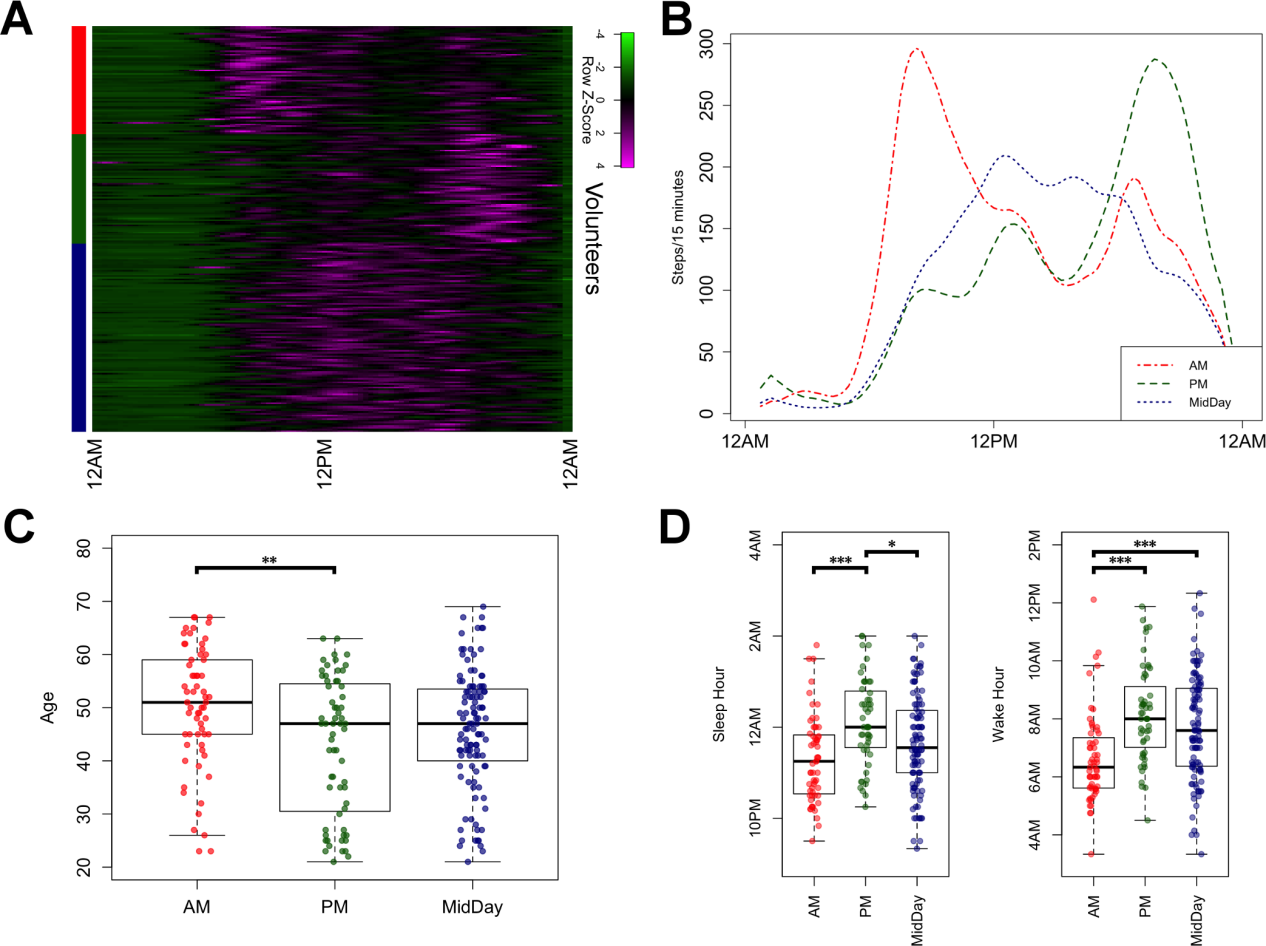
Stratification of volunteers based on wearable-derived activity metrics. (A) Heatmap showing activity profiles of study subjects over a 24-hour period, grouped by cluster (red = AM, green = PM, blue = MidDay). (B) Average activity profiles of the AM, PM, and MidDay clusters, respectively. (C) Boxplots showing the age distribution of each cluster. (D) Distribution of sleep and wake times for each cluster. The code to generate this figure can be found in S2 Data. Asterisks denote significance of Tukey’s range test between cluster pairs. * = *p* < 0.05; ** = *p* < 0.01; *** = *p* < 0.001.

Because volunteers comprised males and females of varying age groups ranging from 21 to 69 years, we sought to determine whether there were clusters of volunteers defined by common activity patterns. We obtained per-volunteer daily activity profiles by averaging step counts from multiple days by time of day. These daily profiles were then clustered using unsupervised k-means clustering (k = 3), with Pearson correlation as a distance measure (Fig 1A). The 3 resulting clusters, of sizes 62, 63, and 108, respectively, showed distinct differences in terms of peak activity periods (Fig 1A). The first (AM cluster) showed peak activity in the morning, whereas the second (PM cluster) showed peak activity in the evening. A third cluster (MidDay cluster) showed a more even distribution of activity, peaking in midday (Fig 1B).

To further characterize these activity clusters, we compared the distribution of subject age and gender among the 3 clusters. There was no significant difference in gender composition among the clusters, and the average ages of subjects in the clusters were 50 (AM), 43 (PM), and 46 (MidDay) years (Fig 1C). Mean age among the clusters was significantly different (one-way ANOVA, *p* = 0.002), with the AM cluster having older subjects compared with the PM cluster (Tukey’s test, *p* = 0.002).

We next considered sleep tracking data and characterized sleep and wake times across the clusters. On average, sleep tracking data revealed that our volunteers spent 6 hours and 57 minutes asleep each day, which is consistent with other studies [18]. Average sleep times for the AM, PM, and MidDay clusters were at hours 23:18, 00:07, and 23:40, respectively, whereas average wake times were at hours 06:39, 08:10, and 07:43, respectively (Fig 1D). There was a significant difference in sleep times among the clusters (one-way ANOVA, *p* = 1.3 × 10^−4^), with the AM cluster going to bed earlier than the PM cluster (Tukey’s test, *p* = 7.18 × 10^−5^) and the PM cluster having a later sleep time compared with the MidDay cluster (Tukey’s test, *p* = 0.019). Similarly, wake times were different between clusters (one-way ANOVA, *p* = 8.98 × 10^−6^), with the AM cluster waking up earlier compared with both the PM cluster (Tukey’s test *p* = 1.47 × 10^−5^) and the MidDay cluster (Tukey’s test *p* = 3.80 × 10^−4^). The significant disparities in age and sleep timing between the AM and the PM clusters reflect lifestyle differences that could, in part, be explained by previous reports of an advance in circadian timing with aging [19].

### Wearable metrics are associated with clinical risk markers

One key aim of this study is to characterize the relationship between wearable metrics and clinical parameters of relevance to CVMD risk. To that end, volunteers had various clinical parameters measured in the clinic upon recruitment, including BMI, WC, systolic blood pressure (SBP), diastolic blood pressure (DBP), as well as fasting levels of total cholesterol (TotalChol), high-density lipoprotein (HDL), low-density lipoprotein (LDL), triglycerides (TGs) and fasting blood glucose (FBG). Wearable metrics of interest that were evaluated against these clinical parameters comprised DailySteps and RestingHR. For comparison, we also evaluated clinically measured RHR values (ECG_HR), as well as questionnaire-derived activity scores (General Practice Physical Activity Questionnaire [GPPAQ]).

After categorizing our volunteers according to commonly used clinical thresholds (see Materials and methods), we used logistic regression to determine the extent to which wearable metrics are associated with clinical risk markers. For models using DailySteps, adjustments were made for age, BMI, and gender as well as interaction between gender and steps in order to account for known gender-specific differences in metabolism and response to chronic exercise [20,21]. For models using RestingHR, age and gender were included as independent covariates.

**Fig 2:**
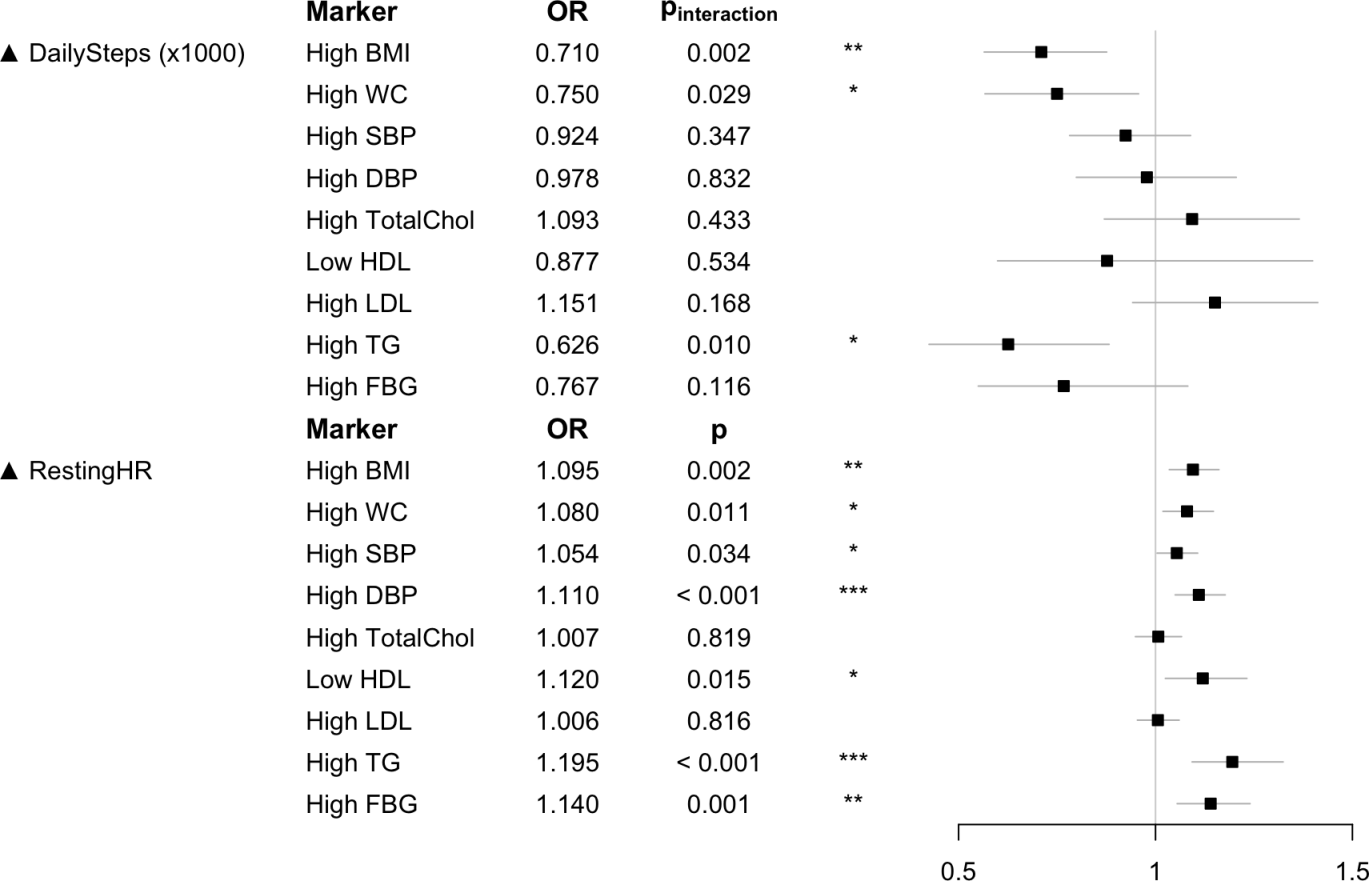
Associations between wearable data (DailySteps and RestingHR) and CVMD risk markers. The forest plot shows the effect and significance of wearable metrics as predictors for clinical risk markers. For steps, the OR is for each additional 1,000 steps. For RestingHR, the OR is for each additional bpm. Details of models used in the logistic regressions and the thresholds used to define the clinical features are provided in the Materials and methods section. *p*-Values and ORs for DailySteps are for interactions between gender and steps, with the female gender being the reference level. The code to generate this figure can be found in S2 Data. bpm, beats per minute; BMI, body mass index; CVMD, cardiovascular and metabolic disorder; DailySteps, average daily steps; DBP, diastolic blood pressure; FBG, fasting blood glucose; HDL, high-density lipoprotein; LDL, low-density lipoprotein; OR, odds ratio; RestingHR, wearable-derived RHR; RHR, resting heart rate; SBP, systolic blood pressure; TG, triglyceride; TotalChol, total cholesterol; WC, waist circumference.

We found that RestingHR is a better predictor compared with DailySteps (Fig 2, S1 Table). Whereas RestingHR was significantly associated with 7/9 clinical markers, DailySteps was only significantly associated with lower odds of having high BMI, WC, and TG values in more active males. For instance, male subjects benefitted more from taking more steps per day in terms of reduced risk of obesity (high BMI) compared with their female counterparts (odds ratio [OR] 0.710; p_interaction_ = 0.002; Fig 2). In contrast, questionnaire-based activity score (GPPAQ) was not significantly associated with any clinical parameters (S1 Table).

Next, we compared RestingHR to gold standard clinical RHR (ECG_HR). For clinical markers that were significantly associated with either measure, RestingHR achieved more significant *p*-values than ECG_HR in all clinical markers except for SBP and DBP, probably due to ECG_HR, SBP, and DBP all being measured during the same clinic visit (S1 Table). This suggests that RestingHR is comparable to gold standard ECG_HR in associating with CVMD risk makers.

### Exercise-induced cardiac remodeling can be identified through wearable-inferred physical activity levels

**Fig 3:**
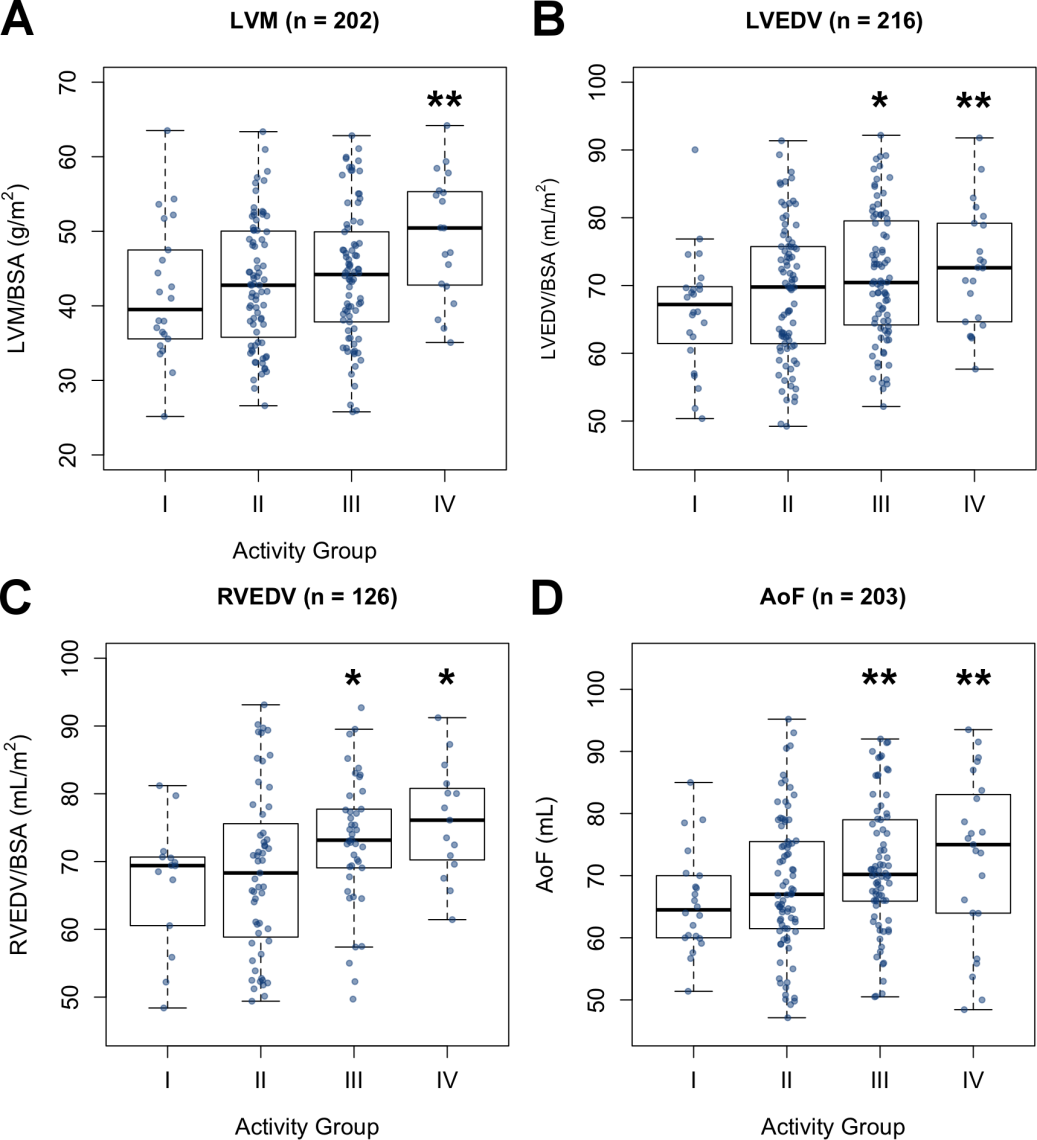
Relationship between wearable-derived physical activity and cardiac parameters. Distribution of (A) LVM, (B) LVEDV, (C) RVEDV, and (D) AoF values across the 4 activity categories (Cat I–Cat IV). The code to generate this figure can be found in S2 Data. Asterisks denote significance of activity category as a GLM predictor with reference to Cat I. * = *p* < 0.05; ** = *p* < 0.01. AoF, aortic forward flow; BSA, body surface area; GLM, generalized linear model; LVEDV, left ventricular end-diastolic volume; LVM, left ventricular mass; RVEDV, right ventricular end-diastolic volume.

A portion of the volunteers underwent CMR imaging. We therefore sought to determine whether wearable-derived metrics could be used in the analysis of cardiac imaging data. In particular, we were interested in whether wearable-derived physical activity was correlated with cardiac remodeling because recent work done using activity questionnaires had indicated that exercise-induced cardiac remodeling (EICR; also known as athlete’s heart) is not exclusive to athletes but can also occur in moderately active individuals [22]. We considered cardiac parameters associated with EICR, namely left ventricular mass (LVM; *n* = 202), left ventricular end-diastolic volume (LVEDV; *n* = 216), right ventricular end-diastolic volume (RVEDV; n = 126), and aortic forward flow (AoF; *n* = 202). The relationship between wearable-derived activity and cardiac parameters was assessed using multiple linear regression adjusting for age, gender, and SBP (covariates and indexing methods used are described in the Materials and methods section). Because cardiac remodeling is more pronounced at the more extreme end of physical activity levels, we also considered DailySteps binned with cutoffs at the 10th, 50th, and 90th percentiles to produce 4 categories (Categories I–IV). We found that both continuous and categorical step counts were significant predictors for all 4 cardiac parameters (Fig 3, S2 Table). After adjusting for covariates, DailySteps (x1,000) was a significant predictor for LVM (β = 0.353; *p =* 0.012), LVEDV (β = 0.386; *p =* 0.010), RVEDV (β = 0.617; *p =* 0.003), and AoF (β = 0.466; *p =* 0.003). We next determined the risk of having abnormally high LVM (indexed to body surface area [BSA]) among our very active volunteers because this is the characteristic most associated with EICR. Those in the upper quartile of DailySteps were more likely to exceed upper population-matched reference limits [23] compared with other volunteers (OR 3.239; CI 1.133–9.276; *p =* 0.026). We also considered RestingHR as a predictor for these cardiac parameters and found it only significant for LVEDV, RVEDV, and AoF but not for LVM (S2 Table). In summary, the integration of wearable activity metrics and cardiac imaging data can reveal relationships between exercise and cardiac remodeling in normal individuals.

### Wearable metrics identify sphingolipid species associated with physical activity and insulin resistance

A subset of volunteers (*n* = 112) underwent serum sphingolipid profiling to determine the abundance of various species of circulating sphingolipids, namely ceramides, sphingomyelins, lactosylceramides, and glucosylceramides. Because circulating sphingolipids, especially ceramides, have been shown to be correlated with cardiorespiratory fitness and exercise [24–26], we sought to determine whether wearable-derived physical activity can contribute to the discovery of relationships between activity and sphingolipid abundance.

**Fig 4:**
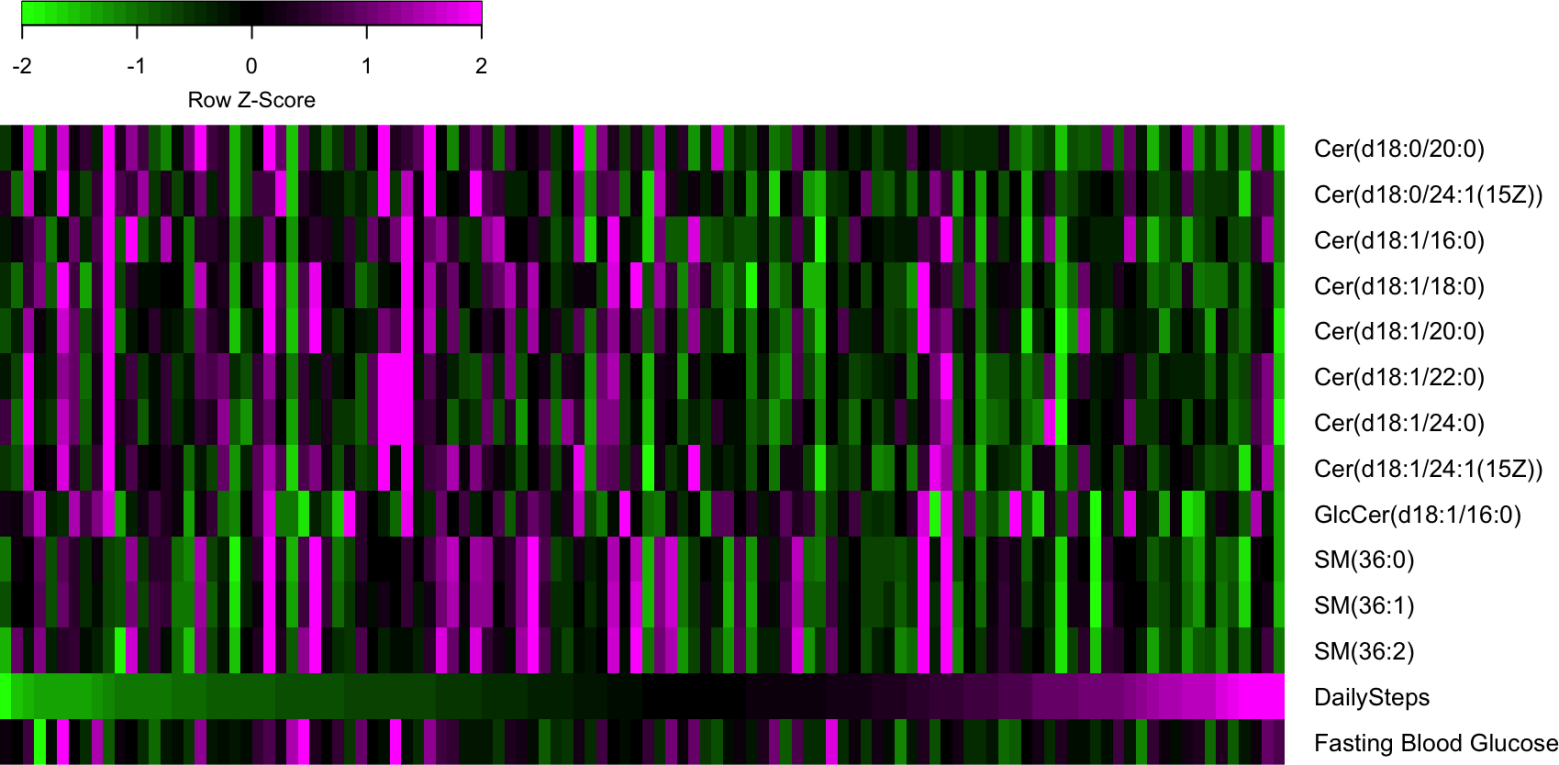
Wearable-derived activity and sphingolipid abundance. Heatmap showing the abundance of sphingolipids that are significantly associated with DailySteps. Columns represent volunteers ordered by increasing DailySteps. For comparison, values of FBG and DailySteps are shown. All values are z-score normalized by row. The code to generate this figure can be found in S2 Data. Cer, ceramide; DailySteps, average daily steps; FBG, fasting blood glucose; GlcCer, glucosylceramide; SM, sphingomyelin.

Using multiple regression to account for age, gender, and BMI, we identified 12 sphingolipids (Table 2, Fig 4) that were significantly associated with DailySteps (*p* < 0.05), 8 of which had a false discovery rate (FDR)–adjusted *p*-value less than 0.1. All significant sphingolipids were negatively associated with DailySteps. Of these, the specific sphingolipids most significantly (*p* < 0.01; q < 0.1) associated with DailySteps included Cer(d18:1/18:0), Cer(d18:1/20:0), and Cer(d18:1/24:1(15Z)), ceramides previously reported to be negatively correlated with cardiorespiratory fitness as measured by peak oxygen consumption in volunteers [25]. We also identified associations among precursor dihydroceramides Cer(d18:0/20:0) and Cer(d18:0/24:1(15Z)), which have been linked to obesity [24,27]. Together, this suggests that wearable-derived physical activity is capable of identifying relationships between lifestyle and serum ceramide abundance. Apart from ceramides, we identified novel associations among several sphingomyelins (SM(36:0), SM(36:1), SM(36:2)) and a glucosylceramide (GlcCer(d18:1/16:0)), all of which were lower in more active subjects. We then compared the abundance of these activity-associated sphingolipids with FBG levels and found that 5 of them were also positively associated with FBG (*p* < 0.05). These included ceramides (Cer(d18:1/18:0), Cer(d18:1/20:0)) and sphingomyelins (SM(36:0), SM(36:1), SM(36:2)) (Table 2). In line with our findings, levels of ceramides (Cer(d18:1/18:0), Cer(d18:1/20:0))—as well as sphingomyelin SM(36:1) in plasma and skeletal muscle—have been reported to be correlated with insulin resistance [26,28–31]. This analysis was also repeated using RestingHR as a metric; however, no significant associations were identified after accounting for multiple testing. These results suggest that activity metrics from consumer-grade wearables are sufficiently accurate to yield biologically relevant insights from lipidomics datasets.

**Table 2:** List of sphingolipids significantly associated with DailySteps.

Sphingolipid	DailySteps (x1,000)		FBG	
	*p*-value	β	*p*-value	β
Cer(d18:1/20:0)^*^	0.002	−0.073	0.031	0.112
**Cer(d18:0/20:0)**	0.004	−0.066	0.434	0.044
**Cer(d18:1/24:1(15Z))**	0.004	−0.067	0.391	0.045
Cer(d18:1/18:0)^*^	0.005	−0.071	0.024	0.112
**Cer(d18:0/24:1(15Z))**	0.009	−0.062	0.502	0.035
**Cer(d18:1/16:0)**	0.013	−0.061	0.575	0.028
**Cer(d18:1/22:0)**	0.014	−0.060	0.055	0.095
SM(36:0)^*^	0.015	−0.056	0.024	0.123
Cer(d18:1/24:0)	0.023	−0.053	0.188	0.067
SM(36:1)*	0.027	−0.051	0.045	0.109
GlcCer(d18:1/16:0)	0.043	−0.043	0.066	−0.113
SM(36:2)*	0.048	−0.045	0.021	0.125

Associations with DailySteps are adjusted for age, gender, and BMI. Sphingolipids significant after FDR (false discovery rate) correction (q < 0.1) are highlighted in bold, whereas those that are also significantly associated with FBG levels are marked with an asterisk (*). The code to generate this table can be found in S2 Data.

Abbreviations: BMI, body mass index; Cer, ceramide; DailySteps, average daily steps; FBG, fasting blood glucose; FDR, false discovery rate; GlcCer, glucosylceramide; SM, sphingomyelin.

## Discussion

The data we presented above show that even short-duration wearable studies can provide value to biomedical science, particularly in cardiovascular and lipidomics research. Our analysis of time series activity data shows that wearable metrics can stratify a cohort into behavioral clusters with distinct characteristics. This approach can assist researchers seeking to correlate lifestyle physical activity with health outcomes.

Our characterization of the relationships between wearable metrics and CVMD markers can also be used to inform future wearable studies. Specifically, we found that RHR metrics are superior to step-based ones in terms of association with CVMD markers. However, this was not the case for the cardiac imaging and lipidomics data. This is likely attributable to RHR being a strong determinant of CVMD risk [5]. Conversely, unlike RHR, the relationship between activity and CVMD risk is less straightforward because it is subject to many confounders not captured in this study, of which diet is likely to be a key factor. Such relationships may only become apparent with larger sample sizes and diverse cohorts [32].

Despite rising adoption of wearables for personal activity and fitness tracking, the translation of wearable metrics into actionable health insights remains a challenge. Our results show that, with respect to CVMD risk, RHR values from wearables are equivalent to—if not better than—clinical RHR measurements. With wearables, users can continuously monitor RHR, enabling early detection of deviations in RHR that herald changes to CVMD risk markers such as weight gain and hypertension [33]. This may improve upon the status quo, whereby individuals are typically only made aware of changes to health status and disease risk during infrequent health checkups. Notably, the correlation between wearable metrics and fasting glucose suggests that wearables may provide an early indication of increased diabetes risk, which is vital given that a large number of Singaporeans have undiagnosed diabetes [34]. From a healthcare research perspective, continuous RHR tracking may become essential in long-term studies tracking lifestyle and health parameters because it could provide a high-resolution view of how changes in lifestyle impact cardiovascular health. Such insights, potentially emergent from large-scale efforts such as Project Baseline (http://www.projectbaseline.com), will also help formulate ways to present wearable data to users in ways that promote compliance with healthy lifestyle behaviors and facilitate early detection of disease states.

Our findings on the utility of wearables in analyzing CMR data and identifying individuals at risk of having abnormally high LVM have clinical and research implications. EICR is a benign adaptation to increased cardiovascular load resulting from exercise [35], although there are conflicting reports of malignant adaptation to excessive exercise [36,37]. Initially thought to be exclusive to competitive athletes, a recent population study found that 14% of individuals in the most active category met imaging criteria for left ventricular hypertrophy [22]. Overlapping features between EICR and hypertrophic cardiomyopathy pose a risk of overdiagnosis by clinicians faced with abnormal findings in nonathletic patients, particularly at a time when regular exercise is heavily promoted to the public [35,38]. Apart from increased awareness of this phenomenon by clinicians, wearable metrics can play a role in differentiating between pathologic and physiological remodeling.

CMR is the preferred imaging modality for population-scale health studies (e.g., UK Biobank [*n* = 100,000] [39], German National Cohort [*n* = 30,000] [40], Canadian Partnership for Tomorrow [*n* = 10,000] [41]) because it avoids exposure to ionizing radiation or contrast agents [39]. Our findings hint at a wider role for wearables in population studies aiming to dissect the relationship between lifestyle and cardiac function. The rising number of normal individuals undergoing CMR as part of population-scale studies implies that a non-negligible fraction will be flagged with abnormal CMR findings. Where follow-up of incidental findings is consented and authorized, wearables could thus play a role in reducing the risk of overdiagnosis.

Ceramides are sphingolipids involved in cellular stress response and are linked to pathological conditions such as insulin resistance, obesity, and cardiovascular disease [42]. Furthermore, higher levels of cardiorespiratory fitness are associated with lower plasma ceramide abundance, suggesting that physical activity may play a beneficial role in regulating levels of these molecules. Using wearable-derived activity data, we identified specific sphingolipids associated not only with activity but also with insulin resistance. Among the top activity-associated sphingolipids, 3 ceramides—Cer(d18:1/16:0), Cer(d18:1/18:0), and Cer(d18:1/24:1(15Z))—have been previously shown to predict risk of major adverse events due to cardiovascular disease [43,44] and have been included in clinical laboratory tests [45], showing that our approach can identify lifestyle-modifiable sphingolipids linked to health outcomes. Whereas previous studies investigating relationships between cardiorespiratory fitness and circulating ceramides have been interventional [25,46] (i.e., using exercise training programs or graded treadmill test), we found that analysis of baseline wearable-derived activity levels can also provide insights into such relationships. Future studies, including population cohorts, could be conducted using commodity wearable devices, ultimately enabling more data to be collected while reducing study complexity.

One limitation of this study is the relatively short duration of the tracking periods, thus compromising power to detect associations between activity and CVMD markers. Our cardiac imaging and lipidomics analyses suggest that longer tracking periods, particularly feasible when volunteers share data from their personal devices, will prove to be even more useful. Additionally, volunteers recruited into this cohort may be enriched for those with a higher level of regard for their health and well-being. There was limited examination of time series (as opposed to summary)–wearable data. Activity cluster membership (with the AM cluster as reference) was tested as a predictor for all the association analyses in this study; however, apart from LVEDV (*p*_PM_cluster_ = 0.071; *p*_MidDay_cluster_ = 0.014) and AoF (*p*_PM_cluster_ = 0.467; *p*_MidDay_cluster_ = 0.025) in the cardiac imaging data, there were no other significant associations. Further studies on the utility of features derived from the time series data (e.g., HR variability, HR recovery, activity intensity) are therefore warranted.

In summary, we have characterized in a sizeable cohort the relationship between wearable metrics and a wide range of volunteer phenotypes including lifestyle patterns, demographics, CVMD clinical markers, cardiac imaging, and serum sphingolipid profiles. Our findings show that apart from fitness tracking, consumer-grade wearables can play a role in both basic and clinical research. Such wearables could also provide a low-cost means for early detection of changes in an individual’s personal CVMD risk profile, potentially resulting in more timely detection and intervention of CVMDs.

## Materials and methods

### Study volunteers and ethics statement

The SingHEART/Biobank study was established at the NHCS to characterize normal reference values for various cardiovascular and metabolic disease-related markers in Singaporeans. Normal volunteers were enrolled into this study using a protocol and written informed consent form approved by the SingHealth Centralized Institutional Review Board (ref: 2015/2601). The volunteers underwent comprehensive profiling in the following areas: (1) activity tracking using the Fitbit Charge HR wearable sensor, (2) physical activity and lifestyle questionnaire, (3) CMR imaging, (4) serum sphingolipid profiling, (5) fasting lipid and glucose panel, and (6) assessment of clinical parameters (e.g., HR, blood pressure, BMI). A total of 233 volunteers were included in this study after evaluation for completeness of activity tracking data (details below) and removal of subjects with extreme outlier activity metrics (potentially due to improper wearable usage). Inclusion criteria were as follows:

1. Aged between 21 and 69 years.
2. No personal medical history of myocardial infarction (MI), coronary artery disease (CAD), peripheral arterial disease (PAD), stroke, cancer, autoimmune/genetic disease, endocrine disease, diabetes mellitus, psychiatric illness, asthma, or chronic lung disease and chronic infective disease.
3. No family medical history of cardiomyopathies.

### Activity tracking

Volunteers were issued a Fitbit Charge HR wearable activity tracker to be worn over a course of 5 days (e.g., typically Monday–Friday). However, because the first and last days of the study tended to be partial days, the average yield for each study was 3 days of complete tracking (defined as ≥20 hours with steps and HR data). Data for each subject were downloaded from the Fitbit website using the “fitbitScraper” package (https://github.com/corynissen/fitbitScraper; NB: This method of data access is now deprecated; the same data can be obtained through the Fitbit API at https://dev.fitbit.com/reference/web-api/quickstart). Step counts were available at 2 levels: intraday step counts in 15-minute intervals and daily totals. Intraday HR data were available at 5-minute intervals, along with confidence levels. Intraday sleep tracking data containing details of each sleep session were also retrieved.

To determine data completeness, we used HR confidence values as an indicator that the subject is wearing the device. HR data points with confidence value of “−1” were considered to be invalid (i.e., device was not worn or was incorrectly worn). First, the HR values table was merged with the steps table by their time points. We then counted the number of hours per day that contained HR data with a valid confidence value. Days with ≥20 valid hours were considered to be complete. Furthermore, days with no intraday step data were excluded. Such events typically arose due to delayed syncing of the device resulting in older data being overwritten.

To determine RestingHR, we calculated the average HR value for time points that met the following criteria: (1) had ≤100 steps take place within the 15-minute interval and (2) had a valid HR value. Day HR was similarly obtained but by restricting to time points between 2 PM and 4 PM, whereas night HR sampled time points between 2 AM and 4 AM.

For DailySteps estimation, we obtained the average sum of steps that took place in data-complete days. We also derived estimated daily steps using an alternative method. Briefly, we obtained step-count data points across data-complete days that were matched with valid HR values. We then calculated estimated daily steps by multiplying the average of these step-count values by 96 (i.e., the number of 15-minute intervals per day). Daily step values derived through both methods were highly correlated (r_s =_ 0.955; *p* < 2.2 × 10^−16^). We thus used DailySteps as a measure of wearable-derived physical activity for the rest of this study.

Sleep tracking data was processed as follows: the amount of sleep for each day was determined by summing the duration of all sleep sessions for that day. An average sleep duration was then obtained from the data-complete days. Sleep hour was determined by calculating the average start time of sleep sessions occurring between 7 PM and 4 AM. Wake hour was determined by averaging the end time of sleep sessions. Only subjects with average sleep duration, sleep hour, and wake hour that were within 2 standard deviations (SDs) from mean values were included for statistical analysis in the study (216/233 subjects).

### Clustering of daily activity profiles

For each volunteer, we obtained a 24-hour daily activity profile in the following manner. First, step-counts were smoothed across sliding windows of 5 time points. Then, step-counts from multiple data-complete days were collapsed into a single average profile by averaging step-counts from the same daily time point. Time points with no valid step counts were filled with zeros, and the daily profile was again smoothed in 5–time point sliding windows. Volunteers were clustered using unsupervised k-means clustering (k = 3) with Pearson correlation as a distance measure.

### Physical activity questionnaire

As part of the study, volunteers answered several questionnaires to ascertain their lifestyle. Physical activity was primarily assessed using the GPPAQ (https://www.gov.uk/government/publications/general-practice-physical-activity-questionnaire-gppaq). Briefly, subjects provide information on amount spent in the last week on the following activities: (1) physical exercise, (2) cycling, (3) walking, (4) housework, and (5) gardening and/or do-it-yourself activity. Additionally, volunteers were asked of the amount of physical activity involved in their occupations, as well as their walking pace. A physical activity index (PAI) was generated based on the amount of physical exercise and/or cycling as well as their occupational activity level. The PAI has four activity levels: Inactive, Moderately Inactive, Moderately Active, and Active. These are treated as numerical scores in this study. In this study, amount of cycling was not factored into PAI derivation in order to facilitate a more direct comparison with wearable-derived activity.

### Clinical parameters

The following measurements were performed on the day of volunteer recruitment. First, weight and height were measured using a SECA703 weighing scale (Seca), whereas SBP and DBP were obtained using an Intellivue MX450 patient monitor (Philips). Volunteers fasted for 8 to 10 hours prior to the recruitment appointment, and blood was drawn for the following tests: (1) lipid and glucose panel and (2) serum sphingolipid profiling. Additionally, the volunteers also underwent a Pagewriter TC30 16-lead ECG test (Philips). HRs were obtained from two separate sources, the blood pressure monitor (“ABPM_HR”) and from the ECG reading (“ECG_HR”).

### Clinical risk markers and thresholds

The following clinical markers were considered in the study: BMI, WC, SBP, and DBP as well as fasting levels of TotalChol, HDL, LDL, TG, and FBG. Thresholds used to define risk levels (S3 Table) are as follows: High BMI (>27.5), High WC (>100 cm for males, >90 cm for females), High SBP (>140 mmHg), High DBP (>90 mmHg), High TotalChol (>6.2 mmol/l), Low HDL (<1 mmol/l), High LDL (>4.1 mmol/l), High TG (>2.3 mmol/l), and High FBG (>6 mmol/l).

### Association tests

Multiple linear regression and logistic regression analyses described in this study were conducted using the GLM (generalized linear model) function in R. For multiple linear regression, a Gaussian error distribution was used, whereas a binomial one was used for logistic regression. When gender was considered as a covariate, the female gender was set as the reference level.

For logistic regression analysis between wearable metrics and clinical parameters, two models were used depending on the metric. For step-based metrics, the model is Clinical_Marker ~ Age + Gender + Metric + Gender × Metric, whereas for RHR-based metrics, the model is Clinical_Marker ~ Age + Gender + Metric. ORs and *p*-values reported for step-based metrics are for the Gender × Metric interaction term. To ensure that results from various metrics are comparable, association analyses were conducted on a subset of subjects (223/233) with valid measurements for all metric types (i.e., DailySteps, RestingHR, ABPM_HR, ECG_HR, GPPAQ score). For logistic regression between activity clusters (AM cluster as reference level) and clinical markers, the following model was used: Clinical_Marker ~ Age + Gender + DailySteps + Gender × DailySteps + ActivityCluster.

### CMR

CMR of the volunteers was performed using either a Magnetom Aera 1.5T (Siemens) or Ingenia 3T (Phillips) scanner under previously described settings [23]. Parameters such as cardiac volumes and mass were analyzed from imaging data using the CMR42 software (Circle Cardiovascular Imaging) and standardized protocols [23]. Four cardiac parameters were considered in this study: LVM, LVEDV, RVEDV, and AoF, with the first three being indexed to BSA according to the Dubois formula [47]. When performing multiple linear regression, adjustment was made for age, gender, and SBP. In the case of AoF, additional adjustment was made for weight and height. Only cardiac parameters with values within 2 SDs from their mean value were included for analysis. Numbers of data points analyzed for each cardiac parameter are as follows: LVM (*n* = 202), LVEDV (*n* = 216), RVEDV (*n* = 126), and AoF (*n* = 203). For logistic regression analysis of abnormally high BSA-indexed LVM against volunteer physical activity, only those of Chinese ethnicity were considered (*n* = 192). Cutoffs for defining abnormal BSA-indexed LVM were obtained from a study of 180 healthy Singaporeans (70 g/m^2^ for males, 50 g/m^2^ for females) [23].

### Lipidomics profiling and analysis

Lipid internal standard mix (Ceramide/Sphingoid Internal Standard Mixture I, Avanti Polar Lipids) of 500 pmol was added to 100 μl of serum in a microcentrifuge tube. After an equilibration period of 30 s, 1.2 ml of HPLC-grade methanol was added to the mixture, followed by vortexing. The mixture was then incubated at 50°C for 10 min, followed by centrifugation to pellet the precipitated protein. The supernatant was then removed and placed in a clean microcentrifuge tube for drying under nitrogen gas. To reconstitute the dried extract, 100 μl of methanol was then used. The reconstituted lipid solution was then separated using a liquid chromatography–mass spectrometry (LC-MS) system (Agilent 1260) and a Thermo Scientific Accucore HILIC column (100 × 2.1 mm; particle size 2.6 μm). Mobile phase A consisted of acetonitrile/water (95:5) with 10 mM ammonium acetate, pH 8.0, and mobile phase B consisted of acetonitrile/water (50:50) with 10 mM ammonium acetate, pH 8.0. For the separation, the column was equilibrated with 100% mobile phase A, increasing to 20% mobile phase B in 5 min, then held for 5 min. The column was then re-equilibrated with 100% mobile phase A for 5 min. Finally, mass spectrometry (MS) and data acquisition were performed using an Agilent 6430 triple-quadrupole mass spectrometer. As data were generated in two batches, normalization was performed within each batch on raw values (measured in pmols) by performing z-score transformation on a per-sphingolipid basis, prior to combining the data. Odd-chain sphingolipids and sphingolipids with missing values in more than 20% of samples were excluded from analysis.

### Software and statistical tests

All statistical analyses in this study were performed using the R statistical environment. Unless otherwise stated, correlations described in this study are Spearman correlation coefficients. Adjustment for multiple testing in the lipidomics analysis was done using the Benjamini-Hochberg FDR method [48].

## Supporting information

S1 Table Results of logistic regression analysis of CVMD risk markers and various metrics, wearable and nonwearable. *p*-Values are shown, with ORs and 95% CIs shown in brackets. Highlighted cells have *p* < 0.05. *p*-Values for DailySteps and GPPAQ score are *p*-values for the Gender × Metric interaction term. Cells with bold values indicate those for which RestingHR performs better compared with ECG_HR. CVMD, cardiovascular and metabolic disorder; DailySteps, average daily steps; ECG_HR, RHR measured by an electrocardiogram test; GPPAQ, General Practice Physical Activity Questionnaire; OR, odds ratio; RestingHR, wearable-derived RHR; RHR, resting heart rate.
(XLSX)

S2 Table Summary of multiple linear regression results for LVM, LVEDV, RVEDV, and AoF using both continuous (DailySteps) and categorical measures of physical activity, as well as RestingHR. Cat I–Cat IV denotes the categorical grouping of subjects based on DailySteps, with Cat I set as the reference. For comparison, results for questionnaire-based (GPPAQ) physical activity scores (as continuous predictors) are shown. Highlighted cells have *p* < 0.05. AoF, aortic forward flow; BSA, body surface area; DailySteps, average daily steps; GPPAQ, General Practice Physical Activity Questionnaire; LVEDV, left ventricular end-diastolic volume; LVM, left ventricular mass; RestingHR, wearable-derived RHR; RHR, resting heart rate; RVEDV, right ventricular end-diastolic volume.
(XLSX)

S3 Table Sources for thresholds used to define clinical risk markers. (XLSX)

S1 Data Characteristics of study participants. (XLSX)

S2 Data All data and R code used in this work. (ZIP)

## Abbreviations

ABPM_HR: RHR measured by an automatic blood pressure monitor
AF: atrial fibrillation
AoF: aortic forward flow
BMI: body mass index
BSA: body surface area
CAD: coronary artery disease
CMR: cardiac magnetic resonance imaging
CVMD: cardiovascular and metabolic disorder
DailySteps: average daily steps
DBP: diastolic blood pressure
DNN: deep neural network
ECG_HR: RHR measured during an electrocardiogram test
EICR: exercise-induced cardiac remodeling
FBG: fasting blood glucose
FDR: false discovery rate
GPPAQ: General Practice Physical Activity Questionnaire
HDL: high-density lipoprotein
HR: heart rate
LC-MS: liquid chromatography–mass spectrometry
LDL: low-density lipoprotein
LVEDV: left ventricular end-diastolic volume
LVM: left ventricular mass
MI: myocardial infarction
MS: mass spectrometry
NHCS: National Heart Centre Singapore
OR: odds ratio
PAD: peripheral arterial disease
PAI: physical activity index
RestingHR: wearable-derived RHR
RHR: resting heart rate
RVEDV: right ventricular end-diastolic volume
SA: sleep apnea
SBP: systolic blood pressure
SD: standard deviation
TG: triglyceride
TotalChol: total cholesterol
WC: waist circumference
